# Supplementary material for: Detection of Fused Genes in Eukaryotic Genomes using Gene deFuser: Analysis of the Tetrahymena thermophila genome
Source: BMC Bioinformatics. 2011 Jul 11;12:279. doi: 10.1186/1471-2105-12-279 (PMC3143110; doi:10.1186/1471-2105-12-279)
Supplement: Additional file 1 — Results of Gene deFuser for the Tetrahymena thermophila genome. This zip file contains the raw results of the analysis of the Tetrahymena genome using Gene deFuser. To view the contents, unzip the file and open the Final_Tet.html file in the resulting folder. [file 1471-2105-12-279-S1.ZIP › Results/112.m00150.html]

Gene deFuser -- Results of Job Final\_Tet

 


Gene deFuser

| Home | Retrieve Results | References | Help |
| --- | --- | --- | --- |

Back to Main Results of Job Final\_Tet

# Query Name: 112.m00150

Candidate fusion gene

## Query Sequence:

MKQEQDSSSLNVFQKLAQQLEQNKQFRVIHIDEQKGLFDQIITAENLLSKSHHAIKIISIFDGIDSVSQQKLQDAKKQALFLKNVNHQNIIKYFDEFQIGFNYFIVMEKYEKDLYQLIEDFKNHETEIPKHLLVSFAYQILSAIKHLKQQNRVLIDLSLRNILIDSQNQIKLYDLGIAKQEQEELTSSFILTAFLKRSLFYYPPELLGEFEMSGELNKKNKLKNFLDADIWAFGICLYALAGASQQELSSIKSKGYQKLTSLDKCLNSILSQILTLDPNQRPSILNILDFFEDMRKQIWTSDDSADLYEHFLEKMKDSQWYLSYQFINICCQIQQKNETYWFYLGYTQHQLNFLSEAIKSYQKCLEINPKDDICYYNLGKAYKEKDLLDEAIKSYQKSIEINPKDDDYYNGLGSAYRAKSLLDEAIKSYQKCLEINPKNDSCFYNLGNAYDDKGLLDEAIKSYQKCLEINPKDDICYYNLGNTQKEKGLLDEAIKSYQKSIEINPKDDDYYNGLGSAYKEKGLVDEAIKSYQKCLEINPKDDIYNYNLGNAYDDKGLLDEAIKSYQKSIEINPKNDSCYYNLGIAYKLKGLLDEAIKSYQKCLEINPKNDSCYYNLGIAYKEKGLLDEAIKSYQKSIEINPNDDDYYKGLGNAYKAKGLLDQAIKSYQKCLEINPNNDICYYNLGNTYKEIGLLDETIKSYQKSIEINPKDDDYYYSLGSAYDDKGLLDEAIKSYQKCLEINPKDDICYYNLGKAYKSKGLLDEAITSYQKSIEINPKDDDCYNSLGSAYDDKGLLDEAIQSYQNCLEINPMDDSCYYNLGNTYKEKGLLDEAIRSYQESIEINPENDSCYYNLGIACKSKGLLDKAIQSYQKCLEIHPKNDSCYYNLGKAYKSKGLVDEAIKSYQSSIEINPKVDAYYNSLGNAYKVKGLLDEAIKSYQNCLKINPNYNSCYYKLGQAYKSKGLLDEAIKSYQKYLEINPKNDSCYYNLGLAYKSKGLLDEAIKSYQKCLSLNPNNKNCQKNLEITQGKKK

### Significant Ortholog Group Hits and their Scores:

| N terminus | | C terminus | |
| --- | --- | --- | --- |
| [D] KOG0591 NIMA (never in mitosis)-related G2-specific serine/threonine protein kinase | 9.42848774286981 | [GOT] KOG4626 O-linked N-acetylglucosamine transferase OGT | 216 |
| [R] KOG0597 Serine-threonine protein kinase FUSED | 5.52620864757867 | [D] KOG1126 DNA-binding cell division cycle control protein | 33.4892885445476 |
|  |  | [DO] KOG1155 Anaphase-promoting complex (APC), Cdc23 subunit | 26.3933952093005 |
|  |  | [R] KOG1124 FOG: TPR repeat | 24.1807067039078 |
|  |  | [R] KOG1125 TPR repeat-containing protein | 18.6734705232136 |
|  |  | [R] KOG1129 TPR repeat-containing protein | 11.1469879012243 |
|  |  | [A] KOG1127 TPR repeat-containing protein | 9.05761042010194 |
|  |  | [O] KOG0548 Molecular co-chaperone STI1 | 7.49744726968476 |
|  |  | [U] KOG0547 Translocase of outer mitochondrial membrane complex, subunit TOM70/TOM72 | 5.20431662415082 |

#### Graphs (click to enlarge):

|  |  |
| --- | --- |
| BLAST of Query Sequence | Location of Ortholog Group Hits |
|  |  |

Contact: Andre Cavalcanti\_\_\_\_\_Last Modified September 14, 2010
